# Awareness and access to mass media sources of information about modern family planning methods among women with disabilities in Nigeria: An analysis of 2018 demographic and health survey

**DOI:** 10.3389/fgwh.2022.746569

**Published:** 2022-12-02

**Authors:** Hussaini Zandam, Monika Mitra, Sophie Mitra

**Affiliations:** ^1^ The Lurie Institute for Disability Policy, The Heller School for Social Policy and Management, Brandeis University, Waltham, MA, United States; ^2^ Economics Department and Research Consortium on Disability, Fordham University, Bronx, NY, United States

**Keywords:** modern contraceptives, disability, knowledge, information, disparity

## Abstract

**Background:**

Family planning is the foundation of sexual and reproductive health, and necessary for achieving the Sustainable Development Goals. Yet, the needs of women with disabilities and their access to these services have been neglected for decades, especially in Low and Middle-income Countries. To improve utilization of these services among women with disabilities, they have to be aware and informed about the services. This study was conducted to examine awareness and mass media sources of information on family planning between women with and without disabilities.

**Methods:**

This study used data from the 2018 Nigeria Demographic and Health Surveys (NDHS). Our analytic sample included 26,585 women between 15 and 49 years of age who answered the disability module. We compared demographics and socioeconomic characteristics of women with and without disabilities using the chi-square test for categorical variables. In addition, we conducted logistic regressions to estimate the unadjusted and adjusted odds ratio (with 95% confidence intervals) for level of awareness and mass media sources of information on modern contraceptive methods, using women without a disability as the reference group.

**Results:**

Finding showed that women with disabilities have poor awareness about family planning compared to women without disabilities even after adjusting for all covariates (AOR = 0.42, 95% CI: 0.23–0.76, *P* < 0.05). We also found that women with disabilities are less likely to receive information about family planning from any of the available mass media channels even after adjusting for covariates (AOR = 0.46, 95% CI: 0.22–0.98, *P* < 0.05).

**Conclusions:**

The study revealed that women with disabilities Nigeria have poor awareness about modern family planning methods compared to non-disabled women. They are also less likely to receive information about modern family planning methods compared to non-disabled women. To effectively reach women with disabilities, information barriers must be eliminated, coupled with increased opportunities to access family planning information. Donors, government, and other relevant stakeholders should consider funding inclusive campaigns and explore other mechanisms for disseminating family planning information to women with disabilities.

## Background

Family planning is the foundation of sexual and reproductive health and necessary for achieving the Sustainable Development Goals (1). Sexual and reproductive health allows individuals to achieve desired birth spacing, family size and improve health outcomes for infants, children, women, and families. Specifically, access to contraceptives prevents unsafe sex, abortions, HIV (human immunodeficiency virus), and other sexually transmitted infections, which constitute significant risk health challenges among women (2, 3). Yet, women with disabilities and their access to these services have been neglected for decades because of many reasons including the widespread assumption that people with disabilities are not sexual or sexually active (4–6). There has been a growing recognition of the rights of people with disabilities since the World Report on Disability was published (7) and the United Nations Convention on the Rights of People with Disabilities, which has ratified by several countries (8). The World Report on Disability, for the first time, brought global recognition to the rights of people with disabilities, who are often described as the world's largest minority group. The report officially recognizes, among others, the need for sexual and reproductive health policies and programs to be inclusive of women with disabilities (7).

Nigeria is an important country especially in light of the global contraceptive goal referred to as 120 by 20, which aims to increase access to modern contraceptives for 120 million more women by 2020 (9). With national modern contraceptive prevalence rates staggering at 12.5%, Nigeria has struggled to achieve its country goal of increasing contraceptive uptake by more than 1.5% per year in order to reach Sustainable Development Goal target (10–12). Low uptake of contraceptives in the country has been associated with myriad of factors including cultural and religious factors (13, 14) and lack of adequate knowledge and poor attitudes (15–17). Several initiatives have been implemented to improve contraceptive uptake including awareness campaigns through mass media channels. Studies have shown that improving awareness through mass media can influence people into adopting family planning methods (18–20). For instance, the impact evaluation of the Nigerian Urban Reproductive Health Initiative showed that between 2015 and 2020, awareness campaign through radio, television, and community events significantly increased the use of modern contraceptives from 21.1% to 30.1% in the states the program was implemented (21). Despite the potential of the education and awareness interventions, program deficiencies have also been observed especially in targeting harder-to-reach groups of women (12). A review of program and policy documents on contraception found that women with disabilities were not included in the family planning program design and planning in Nigeria (22–24).

Monitoring disability-inclusive policy and programming require a nationally representative and internationally comparable data to measure and close any existing gaps. The available literature on family planning in sub-Saharan Africa does not account for the disability-related disparities in knowledge and access to modern contraceptives. To address this gap, we analyzed nationally representative data from the Nigeria Demographic and Health Surveys (NDHS) to compare contraceptive knowledge and information sources between women with and without disabilities. We hypothesized that women with disabilities are more likely to report poor knowledge of modern contraceptives and limited access to information on modern contraceptives than non-disabled women. A study on the knowledge and access to information about family planning using an internationally comparable definition of disability and a nationally representative sample will not only contribute to close the gap in research on modern contraceptives among women with disabilities, it may also form the basis for developing inclusive interventions to improve contraceptive knowledge and demand among women with disabilities.

## Methods

We conducted a secondary analysis of the publicly available 2018 Nigeria Demographic and Health Surveys (NDHS) (25). The NDHS is supported by the United States Agency for International Aid (USAID) and provides up-to-date estimates of key demographic, socioeconomic and health indicators in Nigeria including sexual and reproductive health in adults, infant and maternal mortality, child mortality, nutritional status, malaria, and disability status. The NDHS employed a stratified two-stage sample survey design. In the first stage, primary sampling units (PSUs) or enumeration areas (EAs) in urban and rural areas were selected. In the second stage, a random sample of residential dwelling units (DUs) from each PSU was selected for the survey.

The Disability Module is part of the Household Questionnaire offered to all identified household members. Detailed information about survey design, sampling methods, and response rates is available in the NDHS final survey reports (25). The NDHS data are nationally representative of women 15–49 years of age. A total of 41,821 women were interviewed in 2018. Of these, only 26,585 were offered the disability module and formed our analytic sample.

### Measurements

Our dependent variables were as follows: (i) awareness of modern family planning methods; and (ii) access to information about modern family planning methods from mass media sources. The first dependent variable was generated from a questions asking knowledge of contraceptive methods including, lack of knowledge of any method, knowledge of traditional method, folkloric method, and modern methods. Responses were categorized as yes/no question where yes indicates familiarity with any modern contraceptive method (e.g., contraceptive pill, male condom, intrauterine contraceptive device etc.). The second dependent variable was generated from four yes/no questions asking whether the respondent heard or read information about modern family planning methods in the last few 12 months (1) on radio; (2) on TV; (3) in newspaper or magazine, leaflets or brochure; and (4) from mobile public announcement.

### Disability

The primary explanatory variable was disability and was assessed from responses to the Washington Group Short Set of Questions on Disability (WGSS) (26). It is the standard approach to measuring disability in censuses and large surveys producing valid data that is internationally comparable (27). Disability status assessment was based on experience of difficulties by an individual related to: (1) seeing; (2) hearing; (3) walking; (4) remembering; (5); communicating and (6) washing or taking care of self. Possible responses to the questions were as follows: no difficultly; some difficulty; a lot of difficulty; and cannot do at all. We employed the recommendation from the Washington Group on Disability Statistics' analytical guidelines in creating a dichotomous disability categorization (26). Women who reported “a lot of difficulty” or “cannot function at all” to any of the six functional domains were classified as having a disability; women who reported “none” or “some difficulty” were classified as not having a disability (26). Despite the validity of the WGSS, it also has limitations, especially not capturing some types of disabilities such as mental health-related disabilities (28).

### Covariates

Covariates for this study included age (<25 years, 25–34, or 35+ years), education (no education or having at least primary level education), union status (married or single), religion affiliation (Christian or Muslim/traditionalists), employment status (working or not working) and parity (0, 1–2, 3–4, and 5+). Household characteristics included household wealth (poorest or others) and place of residence (urban or rural).

### Statistical methods

We compared demographics and socioeconomic characteristics of women with and without disabilities using the chi-square test for categorical variables. Both dependent variables were analyzed as binary (yes/no) variables. We calculated the proportions for each outcome indicator and compare between women with and without disabilities. Then we conducted logistic regressions to estimate the unadjusted and adjusted ratios (with 95% confidence intervals) for awareness and access of information, using women without a disability as the reference group. We used Stata version 16 for all analyses, applying *svy* commands to account for the complex sampling design of the NDHS, and we used a *P*-value <.05 as the highest level of acceptable significance.

## Results

### Women's characteristics

Table 1 describes the sample and provides bivariate contrasts in sociodemographic and household characteristics between Nigerian women with and without disabilities. In comparison to women without disabilities, women with disabilities are more likely to be older and, have no education, and be from poorer households. Similarly, women with disabilities are less likely to be married and to live in urban areas. However, there was no statistically significant difference in employment status between the two groups of women.

**Table 1:** Demographic characteristics by disability status.

Characteristics	Women with disabilities (159)	Women without disabilities (26, 426)	*P*-value
**Age**			<0.001
<25	18.9	38.1	
25–34	28.9	31.7	
35+	52.2	30.2	
**Education**			0.021
No education	44.1	35.5	
At least primary education	55.9	64.6	
**Marital status**			0.033
Not married	55.7	40.5	
Married	44.3	59.5	
**Religion**			0.041
Christian	63.3	54.9	
Muslim/traditional	36.7	45.1	
**Place of residence**			0.040
Rural	65.8	54.3	
Urban	34.2	45.7	
**Household wealth quintile**			0.044
Poorest quintile	36.5	22.3	
Other quintile	63.5	78.7	
**Employment status**			0.552
Yes	8.90	12.8	
No	91.1	88.2	
**Parity**			
0	27.0	30.9	0.353
1–2	22.1	24.3	
3–4	22.6	22.1	
5+	28.3	22.7	

*P* significant at <0.05. Source: Authors’ calculations based on the Nigeria Demographic Health Survey (NDHS) 2018.

### Awareness and mass media sources of information about modern family planning methods by disability status

Results from comparison of level of awareness and acces to information on modern methods are presented in Table 2. A significantly higher proportion of women without disabilities (91.3%) reported to have awareness of modern methods compared to women with disabilities (77.8%). Similarly, fewer women with disabilities (19.5%) reported receiving information on modern methods from mass media channels (radio, TV, print media and mobile text message) compared to non-disabled women (32.8%).

**Table 2:** Awareness of modern family planning methods and mass media sources of information about modern family planning methods by disability status.

	Women with disabilities (159)	Women without disabilities (26, 426)	*P*-value
**Awareness about modern contraceptive methods**			<0.001
Yes	77.8	91.4	
No	22.2	8.69	
**Sources of information about family planning**			
Radio	17.1	29.2	<0.001
Television	6.92	16.2	<0.001
Print media	3.17	5.50	0.043
Mobile phone	1.50	4.35	0.281
Mass media (any of the sources above)	19.5	32.8	<0.001

*P* significant at <0.05. Source: Authors’ calculations based on the Nigeria Demographic Health Survey (NDHS) 2018.

### Association between awareness and mass media sources of information about modern family planning methods by disability status

Table 3 present results of unadjusted and adjusted odds ratios from bivariate and multivariate analyses for awareness and access to information on modern methods by disability status. Women with disabilities significantly reported poor awareness of modern methods compared to non-disabled women in the unadjusted analyses (OR = 0.39, 95% CI: 0.26–0.57, *P* < 0.05). The difference in level of awareness remained significant even after accounting for other factors (AOR = 0.40, 95% CI: 0.22–0.72, *P* < 0.05).

**Table 3:** Association between disability status and awareness of modern family planning method and receiving information about modern family planning methods.

	Awareness of modern family planning methods				Mass media sources of information about modern family methods			
	Unadjusted Model OR (95% CI)	*P*-value	Adjusted Model AOR (95% CI)	*P*-value	Unadjusted Model OR (95% CI)	*P*-value	Adjusted Model AOR (95% CI)	*P*-value
**Disability status**								
** **With disability	0.39* (0.26–0.57)	<0.001	0.42* (0.23–0.76)	<0.001	0.48* (0.33–0.73)	<0.001	0.46* (0.22–0.98)	0.049
**Age**								
25–34	2.35* (1.33–2.83)	<0.001	1.54* (1.26–1.87)	<0.00	1.67* (1.57–1.78)	<0.001	1.35* (1.18–1.56)	<0.001
35+	2.57* (1.92–3.39)	<0.001	1.07 (0.83–1.39)	0.041	1.80* (1.68–1.92)	<0.001	1.12 (0.92–1.36)	0.254
**Marital status**								
Never married	0.42* (0.39–0.45)	<0.001	0.62* (0.50–0.75)	<0.001	0.95* (0.90–0.99)	0.033	0.81* (0.68–0.96)	<0.001
**Education**								
No education	0.33* (0.31–0.37)	<0.001	0.34* (0.28–0.39)	<0.001	0.36* (0.34–0.37)	<0.001	0.48* (0.41–0.55)	<0.001
**Wealth quintile**								
Poorest	0.38* (0.35–0.41)	<0.001	0.60* (0.52–0.70)	<0.001	0.33* (0.31–0.36)	<0.001	0.65* (0.55–0.76)	<0.001
**Place of residence**								
Rural	0.44* (0.41–0.48)	<0.001	0.61* (0.53–0.71)	<0.001	0.35* (0.33–0.36)	<0.001	0.49* (0.45–0.55)	<0.001
**Employment**								
Unemployed	0.34* (0.25–0.47)	<0.001	0.47* (0.31–0.72)	<0.001	0.63* (0.55–0.74)	<0.001	0.64* (0.52–0.79)	<0.001
**Religion**								
Muslim/traditional	0.35* (0.32–0.38)	<0.001	0.53* (0.45–0.62)	<0.001	0.64* (0.60–0.67)	<0.001	0.94 (0.84–1.05)	0.308
**Parity**								
1–2	3.22* (2.92–3.95)	<0.001	2.52* (2.03–3.12)	<0.001	1.18* (1.12–1.25)	<0.001	0.99 (0.84–1.18)	0.988
3–4	3.20* (2.89–3.94)	<0.001	2.64* (2.02–3.44)	<0.001	1.21* (1.14–1.27)	<0.001	0.87 (0.71–1.07)	0.204
5+	2.63* (2.39–2.89)	<0.001	2.60 (1.93–3.51)	<0.001	0.95 0.89–1.00	0.080	0.74 (0.58–0.94)	<0.001
**Goodness of fit *(χ*^2^)**			<.001				.026	

* *P* significant at <0.05. Source: Authors’ calculations based on the Nigeria Demographic and Health Surveys, 2018.

Similarly, women with disabilities were less likely to receive information about modern family planning methods *via* any mass media channels compared to women without disabilities (OR = 0.48, 95% CI: 0.33–0.73, *P* < 0.05). The difference remained even after adjusting for all covariates (AOR = 0.46, 95% CI: 0.22–0.98, *P* < 0.05). Other sociodemographic variables including age, marital status, education, wealth, location of residence, parity and religion were independently associated with awareness and mass media access to information.

## Discussion

In this secondary analysis of 2018 NDHS data, we found substantial disparities in awareness and access to information on modern family planning methods between disabled and non-disabled women in Nigeria. Approximately one-fourth of Nigerian women with disabilities had no awareness of any modern family planning method compared to only about one-fifth of women without disabilities. Similarly, only about one-fourth of women with disabilities were reported to have received information from mass media sources about modern family planning methods compared to about one-third of non-disabled women.

Though most women in our study had awareness of modern methods, we found relatively low awareness among women with disabilities and among women who were unmarried, younger, uneducated, Muslim/traditionalists, unemployed, and those from rural and poor households. These sociodemographic differences suggest deficiencies in Nigerian information sharing programs reaching women from low socioeconomic backgrounds (12, 29, 30). Our study also finds that more than half of women in our sample, irrespective of disability status, were not exposed to information about family planning from any mass media channel. This is supported by a previous study using the 2013 NDHS data, which found that 63 percent of women were not exposed to family planning information from the media (31). Our study strengthened the limited reach of family planning messaging in the country.

Women with disabilities in our sample were more likely to be older and unmarried which may also influence their perception and demand for family planning information. Women with disabilities often experience multiple concurrent intersections related to gender and disability. These intersections are often complex and multilayered resulting in women with disabilities experiencing a wide range of attitudinal, communication, and physical barriers when accessing and using sexual and reproductive services (32, 33). The widespread perception that people with disabilities are asexual creates barriers for them to access sexual and reproductive health services (34). This means that they don't know as much about sexual and reproductive services including information as their non-disabled peers. Parents, educators, and health professionals are often uncomfortable or unprepared to discuss sexuality and reproduction issues with disabled people, which could be attributed to normative definitions of sexuality, as well as widespread societal devaluation of disability and cultural portrayals of disabled people as asexual beings (32, 33).

This study has shown some of the challenges of family planning among women with Disabilities in Nigeria, which undermines its effort to achieve its family planning Sustainable Development Goal (1). Disabled women's access to contraceptive information and services is underpinned by internationally recognized human rights, including the right to attain the highest level of health and their right to information that will enable them to make responsible and informed choices about their sexual and reproductive health (35). Nigeria having ratified the United Nations Convention on the Rights of People with Disabilities has a legal obligation to rectify this disparity (36). Women and girls with disabilities are the most vulnerable of the largest minority group of people with disabilities (7). They face a greater risk of neglect, exploitation, and violence both within and outside the home (37). Ensuring equitable access to family planning and its proper utilization can help them reap its socioeconomic and health benefits such as empowerment to determine reproduction and autonomy within their households and enhance their earning power (2, 3). The findings of this study highlight the need for an aggressive, accessible, inclusive family planning campaign such as incorporating screen readers, audio transcriptions for video, and sign language interpretation to increase disabled women's knowledge of modern contraceptives and echo decades' old neglect that people with disabilities have been experiencing regarding their sexual and reproductive health (4–6).

This study has several limitations. The NDHS has no information about the onset, cause, severity, and length of disability. Lack of these information may have limited the scope of the data to capture an individual's experience and access to family planning information. The limited sample size of participants who responded to the disability questions clearly under-represented women with disabilities, which may also influence our finding. The limited sample also made it difficult to disaggregate findings by type of disability, which could provide valuable information on the unique challenges faced by women with different type of disabilities. Another limitation is that of accuracy of disability estimates because disability questions are responded by the head of household or a household representative instead of the individuals. The 2018 NDHS data is the first wave to include disability; limiting our ability to infer causality in our findings. Finally, the DHS is a self-reported survey; hence the data is subjected to potential recall bias, and some questions may be affected by social and cultural sensitivity issues. Despite these limitations, the DHS has been used extensively, and findings have been highly relevant to researchers, policymakers, and non-governmental organizations working across various sectors for the sexual and reproductive health of women with disabilities.

## Conclusion

The study revealed that Nigerian women with disabilities have poor awareness and access to information on modern contraceptives. In order to effectively reach women with disabilities, informational barriers must be eliminated coupled with increased opportunities to access family planning information. Donors, government, and other relevant stakeholders should consider funding inclusive campaigns and explore other mechanisms for disseminating family planning information to women with disabilities.

## Data Availability

Publicly available datasets were analyzed in this study. This data can be found here: https://www.dhsprogram.com/data/available-datasets.cfm.
